# *Acinetobacter baumannii* Secretes Cytotoxic Outer Membrane Protein A via Outer Membrane Vesicles

**DOI:** 10.1371/journal.pone.0017027

**Published:** 2011-02-28

**Authors:** Jong Sook Jin, Sang-Oh Kwon, Dong Chan Moon, Mamata Gurung, Jung Hwa Lee, Seung Il Kim, Je Chul Lee

**Affiliations:** 1 Department of Microbiology, Kyungpook National University School of Medicine, Daegu, Korea; 2 Proteome Research Team, Korea Basic Science Institute, Daejeon, Korea; Charité-University Medicine Berlin, Germany

## Abstract

*Acinetobacter baumannii* is an important nosocomial pathogen that causes a high morbidity and mortality rate in infected patients, but pathogenic mechanisms of this microorganism regarding the secretion and delivery of virulence factors to host cells have not been characterized. Gram-negative bacteria naturally secrete outer membrane vesicles (OMVs) that play a role in the delivery of virulence factors to host cells. *A. baumannii* has been shown to secrete OMVs when cultured *in vitro*, but the role of OMVs in *A. baumannii* pathogenesis is not well elucidated. In the present study, we evaluated the secretion and delivery of virulence factors of *A. baumannii* to host cells via the OMVs and assessed the cytotoxic activity of outer membrane protein A (AbOmpA) packaged in the OMVs. *A. baumannii* ATCC 19606^T^ secreted OMVs during *in vivo* infection as well as *in vitro* cultures. Potential virulence factors, including AbOmpA and tissue-degrading enzymes, were associated with *A. baumannii* OMVs. *A. baumannii* OMVs interacted with lipid rafts in the plasma membranes and then delivered virulence factors to host cells. The OMVs from *A. baumannii* ATCC 19606^T^ induced apoptosis of host cells, whereas this effect was not detected in the OMVs from the Δ*ompA* mutant, thereby reflecting AbOmpA-dependent host cell death. The N-terminal region of AbOmpA_22-170_ was responsible for host cell death. In conclusion, the OMV-mediated delivery of virulence factors to host cells may well contribute to pathogenesis during *A. baumannii* infection.

## Introduction

*Acinetobacter baumannii* is an important nosocomial pathogen that causes a variety of human infections, particularly in severely ill patients [1], [2]. Multi-drug resistance or pan-drug resistance to clinically available antimicrobial agents in this organism induces serious therapeutic issues [3], [4]. *A. baumannii* is generally regarded as a low virulent pathogen [2], [3], but the full genome sequencing shows that this organism harbors a remarkable number of putative virulence-associated genes and elements homologous to the *Legionella*/*Coxiella* type IV secretion apparatus [5]. Several virulence determinants, such as biofilm formation [6], [7], adherence and ability to invade host cells [8], [9], as well as iron acquisition [10] and host cell death [11], have been assessed in previous studies. In prior studies, we determined that the bacterial molecules secreted from *A. baumannii* were directly responsible for host cell death [12]. Among the variety of bacterial molecules, outer membrane protein A of *A. baumannii* (AbOmpA) was identified as a potential virulence factor to induce host cell death via both mitochondrial and nuclear targeting [11], [13]. However, the secretion and delivery of AbOmpA to host cells remain to be thoroughly elucidated.

A wide variety of Gram-negative bacterial species have been demonstrated to secrete outer membrane vesicles (OMVs) during bacterial growth [14]. OMVs are spherical nanovesicles with an average diameter of 20–200 nm and are composed of lipopolysaccharides (LPS), proteins, lipids, and DNA or RNA [15]–[18]. Moreover, OMVs produced by pathogenic Gram-negative bacteria harbor toxins and specific virulence factors, including the heat-labile toxins of enterotoxigenic *Escherichia coli* (ETEC) [19], [20], the Shiga toxin of *E. coli* O157:H7 [21], the cytolethal distending toxin of *Campylobacter jejuni* [22], and the Cif protein of *Pseudomonas aeruginosa* [23]. Upon the delivery of virulence factors to host cells, OMVs perform an important function in bacterial pathogenesis without the direct interaction between the pathogens and the host cells.

We demonstrated recently that a clinical isolate of *A. baumannii* DU202 secreted OMVs into the extracellular milieu during *in vitro* growth; additionally, several putative virulence factors were identified in the OMVs of *A. baumannii* DU202 [24], thereby suggesting that *A. baumannii* OMVs may serve to deliver virulence factors to host cells. In this study, we evaluated the direct effects of *A. baumannii* OMVs on host cells, particularly, the secretion of OMVs from *A. baumannii* during *in vivo* infection, the delivery of virulence factors to host cells via OMVs and subsequent cytotoxicity. Here we report that *A. baumannii* OMVs represent a vehicle for the delivery of bacterial molecules to host cells and also that a potential virulence factor AbOmpA enriched in the OMVs contributes directly to host cell death.

## Results

### Secretion of OMVs from *A. baumannii*

**Figure 1:**
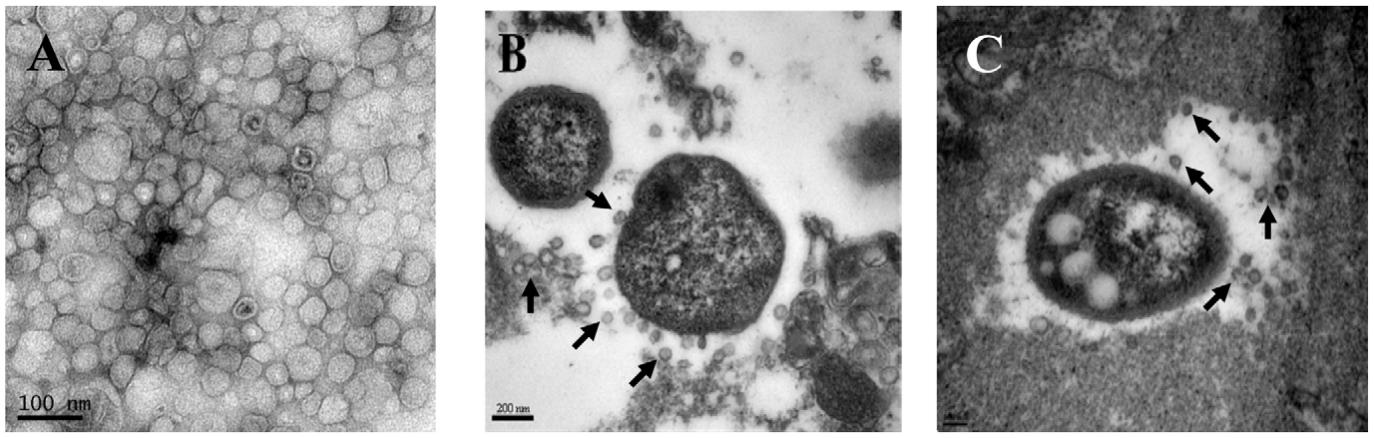
Secretion of OMVs from *A. baumannii* during *in vitro* culture and *in vivo* infection. (A) Transmission electron micrograph of OMVs prepared from *A. baumannii* ATCC 19606^T^ cultured in LB broth for 24 h. (B and C) Secretion of OMVs from *A. baumannii* ATCC 19606^T^ in a murine pneumonia model. Mice were infected with 1×10^7^ CFU of bacteria intratracheally and sacrificed 48 h after bacterial injection. Arrows indicate the OMVs secreted from *A. baumannii*.

We first analyzed the secretion of OMVs from *A. baumannii* ATCC 19606^T^ during *in vitro* culture. Bacteria were cultured in Luria-Bertani (LB) broth and OMVs were collected from the culture supernatants. Transmission electron microscopy (TEM) demonstrated that *A. baumannii* secreted spherical vesicles into the extracellular milieu (Fig. 1A). Next, in order to evaluate the secretion of OMVs from *A. baumannii* during *in vivo* infection, immunocompromised mice were infected intratracheally with 1×10^7^ CFU of *A. baumannii* ATCC 19606^T^ and sacrificed 48 h after bacterial injection. The histological examination demonstrated hemorrhage, necrosis, and infiltration of polymorphonuclear cells in both lung tissues (data not shown). TEM analysis revealed the budding of spherical nanovesicles from bacterial surfaces in the infected lung tissues (Fig. 1B and 1C). These results indicate that *A. baumannii* secretes OMVs during *in vivo* infection and the secreted OMVs are likely to interact with host cells without any direct contact between the pathogens and the host cells.

### Virulence factors packaged in *A. baumannii* OMVs

Proteomic analysis was conducted to identify proteins packaged in the OMVs from *A. baumannii* ATCC 19606^T^. Ultimately, a total of 113 proteins were identified (Table S1). A putative outer membrane protein (A1S_0884) with a molecular mass of 22.5 kDa was detected in the highest abundance, followed by AbOmpA (A1S_2840) with a molecular mass of 38.4 kDa. The cellular localization of proteins identified in the *A. baumannii* OMVs was predicted to occur in the extracellular space (*n* = 1), outer membrane (*n* = 26), periplasmic space (*n* = 8), inner membrane (*n* = 4), cytosol (*n* = 17), and unknown sites (*n* = 57). Virulence-associated proteins, including AbOmpA, CsuA/B (A1S_2218), CsuC (A1S_2215), CsuD (A1S_2214), putative hemolysin (A1S_1321), putative serine protease (A1S_2525), Cu/Zn superoxide dismutase (A1S_3143), fimbrial protein (A1S_1510), bacterioferritin (A1S_3175), RND superfamily transporter (A1S_0116), putative RND type efflux pump (A1S_0009), and putative protease (A1S_2470), were found in the OMVs of *A. baumannii* ATCC 19606^T^.

**Figure 2:**
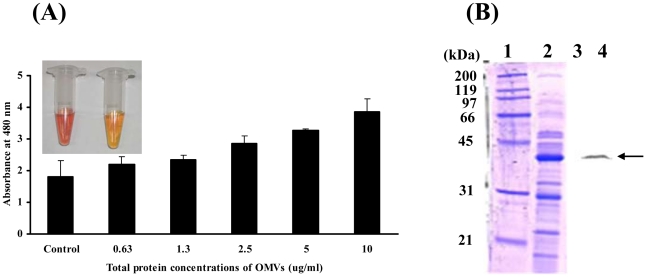
*A. baumannii* OMVs contain biologically active proteins. (A) *A. baumannii* OMVs were incubated with 100 mM nitrocefin in PBS at 30°C for 30 min. Hydrolysis of nitrocefin was monitored at 480 nm using a UV spectrophotometer. Inlet figure: left, brown colorization of nitrocefin in *A. baumannii* OMVs; right, negative control of nitrocefin in PBS. (B) SDS-PAGE of proteins packaged in the OMVs from *A. baumannii* ATCC 19606^T^ (lanes 1 and 2) and its Western blot analysis (lanes 3 and 4). The samples were immunoblotted with a rabbit anti-AbOmpA immune serum. Lanes 1 and 3, molecular weight maker; 2 and 4, OMV fraction. Arrows indicate AbOmpA.

In order to determine whether AmpC β-lactamase (A1S_2367) identified in the *A. baumannii* OMVs were biologically active, the prepared OMVs were incubated with the β-lactamase substrate, nitrocefin. This showed that the β-lactamases associated with *A. baumannii* OMVs degraded nitrocefin, as demonstrated by the colorization of nitrocefin (Fig. 2A). Moreover, the results of Western blot analysis demonstrated that the full length of AbOmpA with a molecular mass of 38.4 kDa was detected in the *A. baumannii* OMVs (Fig. 2B). These results indicate that *A. baumannii* OMVs harbor biologically active virulence factors and enzymes, which can perform diverse biologic processes in host cells.

### Delivery of virulence factors to host cells via OMVs

**Figure 3:**
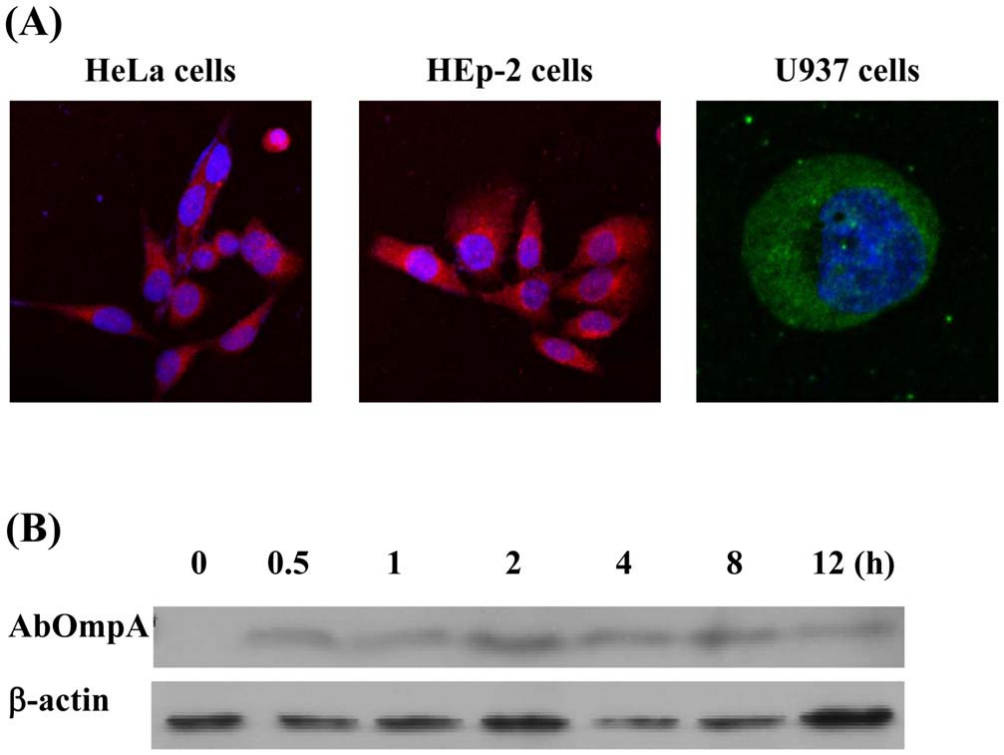
*A. baumannii* OMVs deliver virulence factor AbOmpA into host cells. (A) HeLa and HEp-2 cells were treated with *A. baumannii* OMVs (5 µg/ml of protein concentrations) for 12 h. The cells were fixed, permeabilized, and stained with anti-rabbit AbOmpA antibody, followed by Alexa Fluor® 568-conjugated rabbit IgG (red). DAPI was used to stain the nuclei (blue). Magnification: ×400. The U937 cells were treated with 5 µg/ml of *A. baumannii* OMVs for 4 h and then stained with anti-rabbit AbOmpA antibody, followed by Alexa Fluor® 488-conjugated rabbit IgG (green). Magnification: ×1,260. (B) Western blot analysis of cell lysates. The differentiated U937 cells were treated with *A. baumannii* OMVs (20 µg/ml of protein concentrations) for the indicated times. Cell lysates were separated on 12% SDS-PAGE, transferred to membranes, and immunoblotted with a rabbit anti-AbOmpA immune serum and β-actin antibody.

Based on the results of proteome analysis showing that *A. baumannii* packaged multiple virulence factors into OMVs, we assessed the delivery of virulence factors to host cells via the OMVs. Three human cell lines, HeLa cell, HEp-2 cells, and U937 cells, were treated with OMVs acquired from *A. baumannii* ATCC 19606^T^ for 24 h and the cellular distribution of AbOmpA, a documented OMV protein, was analyzed via confocal microscopy. The cells were treated with 4′,6-diamidino-2-phenyllindole dihydrochloride (DAPI) for nuclear staining and anti-AbOmpA polyclonal antibody, followed by Alexa Fluor® 488 or 568 for AbOmpA. Green or red fluorescence was noted principally in the cytosolic compartments of the cells, but some fluorescence was noted within the nuclei (Fig. 3A). Western blot analysis showed that the full length of AbOmpA appeared within the cells at 30 min and remained there for more than 12 h (Fig. 3B).

**Figure 4:**
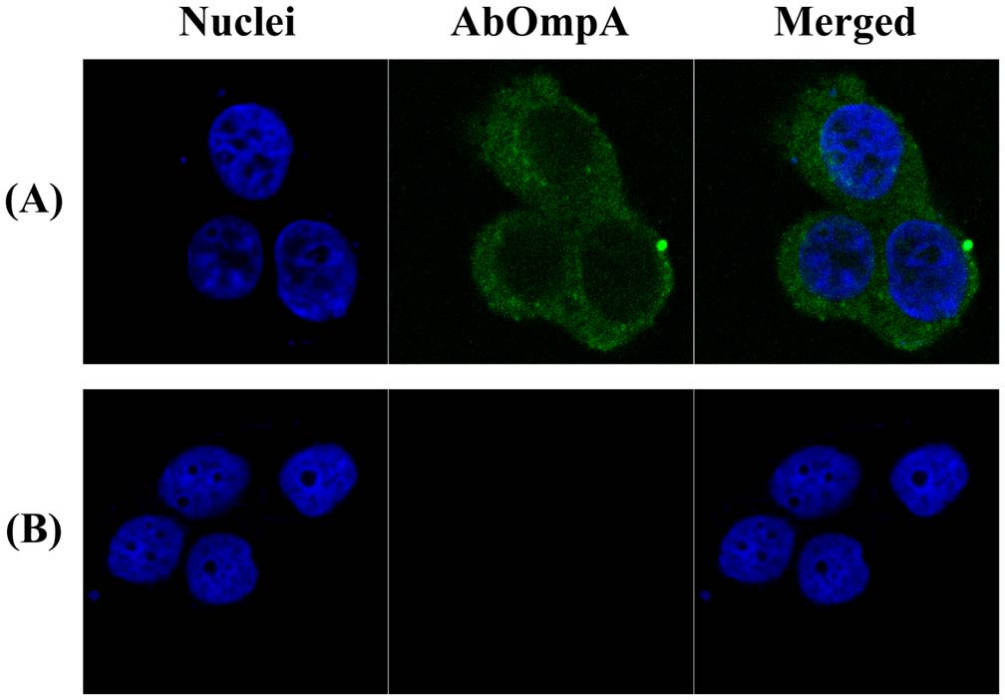
*A. baumannii* OMVs interact with plasma membrane of host cells through a cholesterol-rich membrane microdomain. (A) HeLa cells were treated with OMVs (5 µg/ml of protein concentrations) for 4 h. (B) HeLa cells were preterated with 10 mM MβCD for 45 min and then treated with OMVs for 4 h. The cells were fixed, permeabilized, and stained with anti-rabbit AbOmpA antibody, followed by Alexa Fluor® 488-conjugated rabbit IgG (green). DAPI was used to stain the nuclei (blue). Magnification: ×630.

The OMVs from *E. coli* and *P. aeruginosa* bind to host cells via lipid rafts, after which bacterial effector molecules are translocated into the cytosolic compartment [20], [23]. In an effort to determine whether or not *A. baumannii* OMVs delivered virulence factors to host cells via lipid rafts, HeLa cells were pretreated with a cholesterol-destroying agent, methyl-β-cyclodextrin (MβCD), and subsequently treated with OMVs for 4 h. We used HeLa cells to exclude the involvement of caveolins in the interactions of host cells with OMVs because these cells did not harbor caveolins in the cytoplasmic membrane [25]. AbOmpA was detected in the cytosol of HeLa cells treated with *A. baumannii* OMVs (Fig. 4A), whereas pretreatment of MβCD completely inhibited the cellular localization of AbOmpA in HeLa cells treated with OMVs (Fig. 4B), thereby indicating that a cholesterol-rich membrane microdomain is required for the delivery of virulence factors packaged in the *A. baumannii* OMVs to host cells.

### Cytotoxicity of *A. baumannii* OMVs

**Figure 5:**
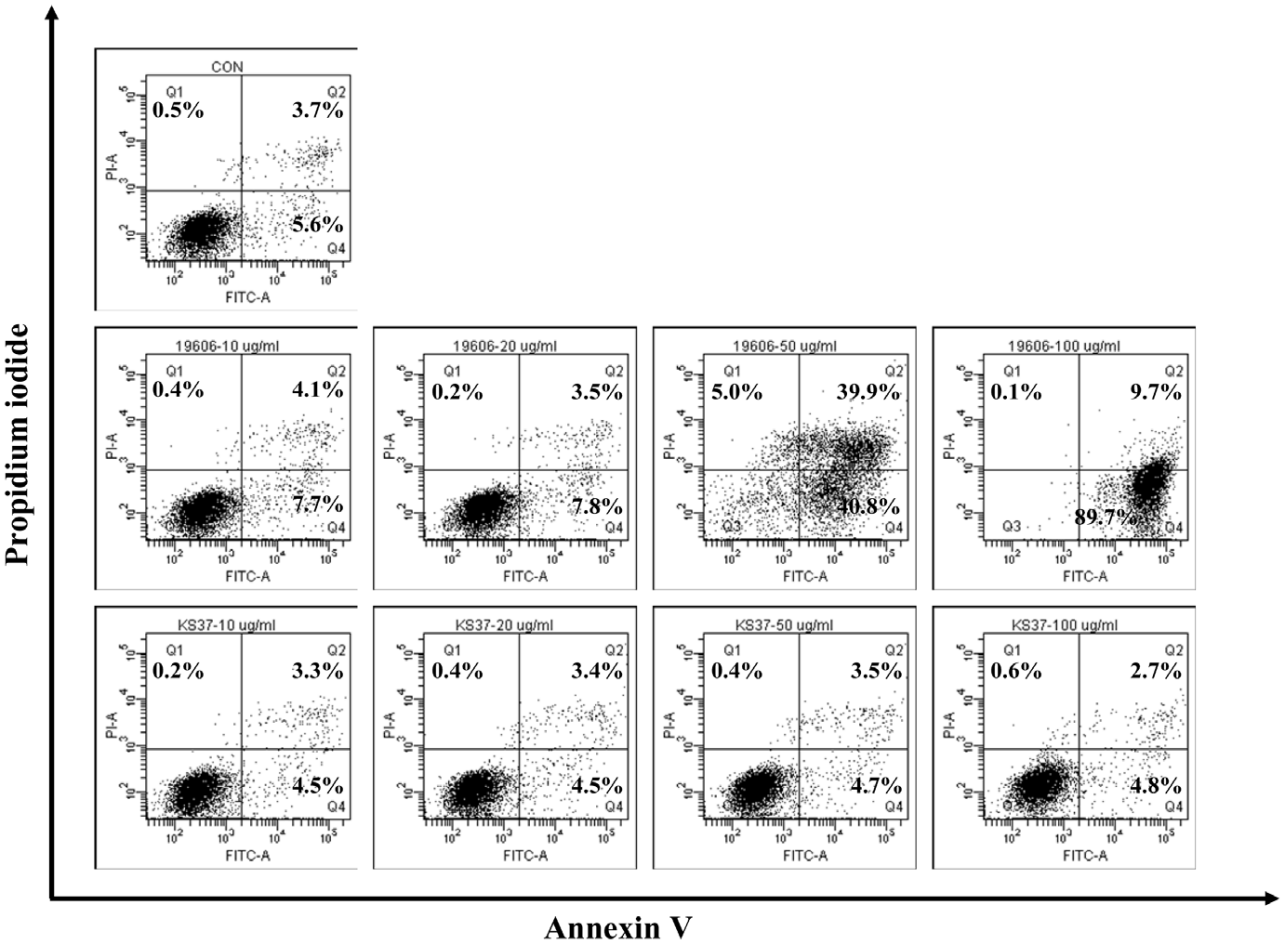
Flow cytometric analysis of cell death induced by the OMVs from *A. baumannii* ATCC 19606^T^ and the Δ*ompA* mutant. The differentiated U937 cells were treated with various concentrations (0, 10, 20, 50, and 100 µg/ml) of OMVs and stained with Annexin V and PI. Upper panel, control cells without OMVs for 24 h. Middle panel, the cells were treated with OMVs from *A. baumannii* ATCC 19606^T^ for 24 h. Lower panel, the cells were treated with OMVs from the Δ*ompA* mutant for 24 h. Representative data from three independent experiments are shown. In the graph, cells in right upper and lower parts are apoptotic cells and cells in left upper part are necrotic cells.

In order to determine whether *A. baumannii* OMVs induced host cell damage, macrophages were treated with various concentrations of OMVs for 24 h and then stained with Annexin V and propidium iodide (PI). The U937 cells were used in this study because macrophages had low threshold concentrations of AbOmpA for cell death as compared with epithelial cells (HEp-2 cells) and fibroblast cells (Cos-7 cells) [13]. Flow cytometric analysis demonstrated no cell death at ≤20 µg/ml (protein concentrations) of OMVs, but 50 and 100 µg/ml of OMVs did induce host cell death (Fig. 5, middle panel). Based on previous studies demonstrating that AbOmpA directly induced apoptotic cell death, OMVs were prepared from the Δ*ompA* mutant and their ability to induce cytotoxicity was compared to that of the OMVs from wild-type *A. baumannii*. The OMVs from the Δ*ompA* mutant did not induce cell death evenly at a concentration of 100 µg/ml (Fig. 5, lower panel). These results suggest that AbOmpA associated with *A. baumannii* OMVs is directly responsible for host cell death.

**Figure 6:**
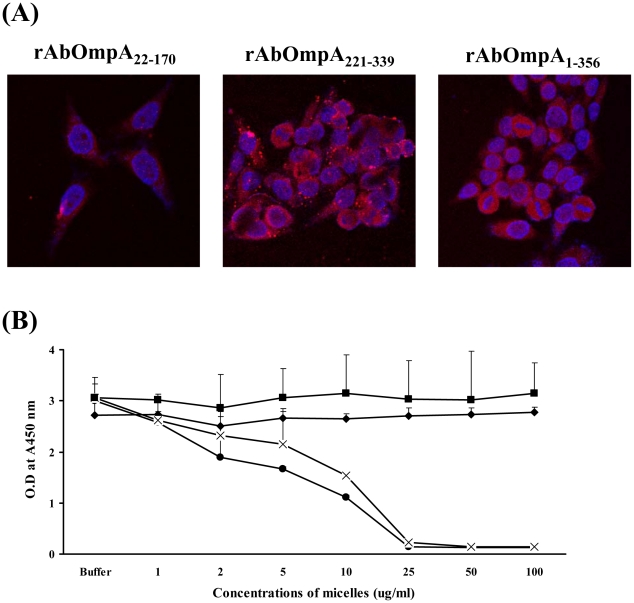
Host cell entry and cytotoxicity of rAbOmpA fragments. (A) HeLa cells were treated with different forms of micelles containing rAbOmpA fragments for 24 h. The cells were fixed, permeabilized, and stained with anti-rabbit AbOmpA antibody, followed by Alexa Fluor® 568-conjugated rabbit IgG (red). DAPI was used to stain the nuclei (blue). The subcellular localization of AbOmpA was observed by confocal microscopy. Magnification: ×400. (B) The differentiated U937 cells were treated with different forms of micelles containing rAbOmpA fragments for 24 h. Cell viability was determined by a WST-1 assay. Untreated control cells (⧫), rAbOmpA_22-170_ (•), rAbOmpA_1-356_ (X), and rAbOmpA_221-339_ (▪).

In order to determine which AbOmpA domains were directly responsible for host cell death, three recombinant proteins, including rAbOmpA_22-170_, rAbOmpA_221-339_, and rAbOmpA_1-356_, were generated and then micelles harboring each rAbOmpA fragment were constructed to insert rAbOmpA proteins into the membrane or lumen according to their hydrophobicity. These micelles were treated to HeLa cells for 24 h, after which the cellular distribution of rAbOmpA was assessed. Three different rAbOmpA proteins were internalized by host cells (Fig. 6A). Next, the cytotoxicity of different rAbOmpA proteins was assessed in the macrophages. Micelles composed of rAbOmpA_22-170_ and rAbOmpA_1-356_ induced cell death at a concentration of ≥5 µg/ml, whereas rAbOmpA_221-339_ did not induce cell death at a concentration of ≤100 µg/ml (Fig. 6B). These results indicate that the N-terminal resides of AbOmpA are responsible for host cell death.

## Discussion

In our previous studies to identify the virulence factors of *A. baumannii*, we demonstrated that AbOmpA induced host cell death via both mitochondrial and nuclear targeting [11], [13]. However, the secretion of AbOmpA from bacteria and its delivery to host cells have yet to be characterized. AbOmpA is a porin that allows for the passing of small solutes in the outer membrane and is one of the most abundant proteins in culture supernatants [11], [24]. Thus, we hypothesized that *A. baumannii* packaged AbOmpA into OMVs and that the OMV-mediated delivery of AbOmpA to host cells induced cytotoxicity. In this study, we identified *A. baumannii* OMVs as an important vehicle for the delivery of AbOmpA to host cells, after which AbOmpA packaged in OMVs induced cytotoxicity. These results help to establish that the previously uncharacterized secretion and delivery pathways of AbOmpA induce host cell death.

Proteome analysis of OMVs from *A. baumannii* ATCC 19606^T^ and a clinical isolate of *A. baumannii* DU202 identified more than 110 proteins derived from the outer membrane, periplasmic space, inner membrane, cytosol, and unknown sites [24]. Twenty-one inner membrane and cytosolic proteins were identified in the OMVs, although OMVs were not likely to contain inner membrane and cytosolic components during the vesicle biogenesis [14], [15]. However, several proteomic studies demonstrated that some inner membrane and cytosolic proteins were also present in the OMV preparations [17], [18], [26]–[28]. A putative outer membrane protein (A1S_0884) with a molecular mass of 22.5 kDa was the most abundant in the proteomic analysis of OMVs (Table S1), but 1-dimentional SDS-PAGE showed the most dense band in the molecular mass of 35–40 kDa (Fig. 2B). This difference is due to the presence of several proteins with a similar molecular mass, such as putative competence protein (ComL) (40.6 kDa), hypothetical protein A1S_1691 (39.2 kDa), AbOmpA (38.4 kDa), β-lactamase (37.3 kDa), hypothetical protein A1S_0505 (36.5 kDa), and putative RND type efflux pump (35.6 kDa). The protein composition of OMVs varied between *A. baumannii* ATCC 19606^T^ and a clinical isolate DU202. Virulence-associated proteins, including AbOmpA, putative RND efflux pump (A1S_0009), bacterioferritin (A1S_3175), and several putative outer membrane proteins, were identified commonly in both *A. baumannii* strains, whereas CsuA/B, CsuC, CsuD, fimbrial protein, and putative hemolysin were identified only in the OMVs from *A. baumannii* ATCC 19606^T^ (Table S1). The protein compositions of the OMVs prepared from a clinical *P. aeruginosa* isolate and from laboratory strain PAO1 differed significantly [29], [30], thus eliciting a different pro-inflammatory cytokine response in macrophages [31]. Accordingly, the strain-specific protein compositions or virulence factor profiles in *A. baumannii* OMVs may result in differences in the virulence of *A. baumannii* strains.

The OMV content is delivered to host cells via either receptor-mediated endocytic pathway or the fusion with host cell plasma membranes [32], [33]. The results of this study demonstrated that a cholesterol-rich membrane microdomain was required for the delivery of AbOmpA packaged in *A. baumannii* OMVs to the cytosol of host cells, thus reflecting the lipid raft-dependent endocytosis of OMVs. Toxins and other vesicular membrane components of OMVs were shown to bind specifically to the receptors within lipid rafts [20], [31]. The heat-labile toxins in ETEC OMVs were bound to monosialoganglioside (G_M1_) [20]. Ellis et al. [31] reported that vesicular LPS and proteins were responsible for the binding of *P. aeruginosa* OMVs to macrophage surfaces and cellular internalization, respectively. Since AbOmpA played a pivotal role in adherence to and invasion of *A. baumannii* in epithelial cells [9], we attempted to determine whether AbOmpA played a role in the interaction of *A. baumannii* OMV with host cells. The Δ*ompA* mutant of *A. baumannii* ATCC 19606^T^ did not express AbOmpA in the outer membrane [11], but the results of proteomic analysis demonstrated the existence of a truncated AbOmpA in the OMVs from the Δ*ompA* mutant (data not shown), thereby suggesting that a truncated AbOmpA may be located in the lumen of OMVs. When HeLa cells were treated with OMVs from the Δ*ompA* mutant, a truncated AbOmpA was detected in the cytosol of host cells. These results indicate that the interaction of *A. baumannii* OMVs with host cells occurred independently of AbOmpA. Future studies will clearly be necessary to identify the bacterial molecules associated with lipid rafts. Moreover, the subsequent trafficking pathway of OMVs following the entry should be clarified to elucidate *A. baumannii* pathogenesis occurring by way of OMV-mediated cytotoxicity.

We previously demonstrated that AbOmpA purified from *A. baumannii* ATCC 19606^T^ and rAbOmpA from *E. coli* caused death in host cells [11], [13], but the cytotoxic domains have yet to be clearly identified. The results of the present study showed that AbOmpA was the most potent cytotoxic molecule in the *A. baumannii* OMVs and the N-terminal 170 residues of AbOmpA were required for the induction of host cell death. A 50 µg/ml (protein concentrations) of OMVs induced host cell death. Because molecular percentage of AbOmpA was determined to be 9.08 based on the proteomic analysis of *A. baumannii* OMVs (Table S1), an approximately 4.5 µg/ml of AbOmpA in the 50 µg/ml of OMVs induced host cell death. Based on the prediction of the tertiary structure of AbOmpA (http://www.pymol.org/), the N-terminal 220 residues of AbOmpA traversed the outer membrane with eight anti-parallel β-sheet segments and four external loops exposed on the bacterial surface. Thus, the N-terminal residues of AbOmpA, which may traverse the vesicular membrane and form external loops in OMVs, induce cytotoxicity. However, the translocation of AbOmpA located in the vesicular membrane to the cytosolic compartment has yet to be characterized.

The contribution of OMVs to bacterial pathogenesis have previously been determined in a variety of pathogenic Gram-negative bacteria, including ETEC [20], uropathogenic *E, coli* [34], *Helicobacter pylori* [35], [36], *Actinobacillus actinomycetemcomitans* [37], *Vibrio cholerae* [38], and *P. aeruginosa* [29]. In this study, we demonstrate that OMVs are an important secretory vehicle for the delivery of virulence factors to host cells in *A. baumannii*. The OMV-mediated cytotoxicity occurring via AbOmpA is a crucial regulator in the induction of host cell death. Further studies of the virulence attributes of each virulence factor packaged in OMVs are expected to provide insights into the association of *A. baumannii* pathogenesis and alterations of host cell biology.

## Materials and Methods

### Bacterial strains and cell culture

*A. baumannii* ATCC 19606^T^ and isogenic Δ*ompA* mutant (KS37) were employed to prepare the OMVs [11]. *E. coli* BL21 (DE3) was employed in the preparation of the rAbOmpA proteins. The organisms were maintained on blood agar plates or MacConkey agar plates, and cultivated in LB broth. HEp-2 cells from laryngeal epithelial cells, HeLa cells from cervical carcinoma, and U937 cells from monocytes were employed in this study. HEp-2 cells and HeLa cells were grown in Dulbecco's modified Eagle medium (HyClone) supplemented with 10% fetal bovine serum (FBS, HyClone), 2 mM _L_-glutamine, 1000 U/ml penicillin G, and 50 µg/ml streptomycin at 37°C in 5% CO_2_. U937 monocytes were differentiated into macrophages for three or four days and matured via the addition of 500 ng/ml of phorbol 12-myristate 13-acetate (Sigma-Aldrich). Macrophages were cultured in RPMI-1640 (Gibco BRL) supplemented with 10% FBS and 2 mM _L_-glutamine at 37°C in 5% CO_2_.

### Purification of OMVs from bacterial culture supernatants

The OMVs from *A. baumannii* were prepared as previously described [18], [39]. In brief, bacteria were grown in 500 ml of LB broth until the optical density at 600 nm (OD_600_) reached 1.0 at 37°C with shaking. Bacterial cells were removed via 15 min of centrifugation at 6,000 x g at 4°C. The supernatants were filtered through a 0.2 µm vacuum filter to remove residual cellular debris. The samples were concentrated via ultrafiltration with a QuixStand Benchtop System (GE Healthcare) using a 100 kDa hollow fiber membrane (GE Healthcare), which could exclude molecules with a molecular mass of 100 kDa in the samples. The OMVs were collected via 3 h of ultracentrifugation at 150,000 x g at 4°C and resuspended in phosphate-buffered saline (PBS). The protein concentration was then determined using a modified BCA assay (Thermo Scientific). The purified OMVs were checked for sterility and stored at −80°C.

### Production of rAbOmpA and micelle formation

The *ompA* gene was amplified from *A. baumannii* ATCC 19606^T^ and cloned into the pET28a expression vector (Novagen) [13], [40]. The recombinant proteins were overexpressed in *E. coli* BL21 (DE3) and loaded onto a HiTrap™ FF column (Amersham Biosciences) to elute His-tagged rAbOmpA. The rAbOmpA was incubated with endotoxin removal resin (Sigma-Aldrich) for the removal of LPS. The protein concentration was determined using a modified BCA assay (Thermo Scientific). In order to construct micelles, the purified rAbOmpA proteins, rAbOmpA_1-356_ and rAbOmpA_22-170_, were incubated with 05% n-dodecyl-N,N-dimethylamine-*N*-oxide in a buffer consisting of 20 mM Tris-HCl (pH 8.0) and 100 mM NaCl. The hydrophilic rAbOmpA_221-339_ was mixed with 20 mM Tris-HCl (pH 6.8) and 100 mM NaCl.

### Identification of proteins in *A. baumannii* OMVs

One-dimensional electrophoresis-liquid chromatography-tandem mass spectrometry (1-DE-LC-MS/MS) was performed to identify proteins packaged in the *A. baumannii* OMVs as described previously [24], [41]. Proteins were separated via 12% sodium dodecyl sulfate-polyacrylamide gel electrophoresis (SDS-PAGE) and in-gel digested. The protein digests were resolved in 15 µl of 0.02% formic acid in 0.5% acetic acid, and the samples were concentrated on a MGU30-C18 trapping column (LC Packings) and a nano-column (C18 reverse-phase column, Proxeon) at a flow rate of 120 nl/min. The peptides were eluted by 0–65% acetonitrile for 80 min. All MS and MS/MS spectra in the LCQ-Deca ESI ion trap mass spectrometer were acquired in data-dependent mode. The MS/MS spectra were searched using MASCOT software (Matrix Science, Inc.) using the genome data of *A. baumannii* ATCC 17978^T^ from NCBInr (http://www.ncbi.nlm.nih.gov/) and the decoy sequence database. The exponentially modified protein abundance index (emPAI) was generated using MASCOT software [42].

### Western blot analysis

Cells were treated with OMVs (20 µg/ml of protein concentrations) for the indicated time periods and then were lysed in lysis buffer (10 mM Tris pH 7.4, 5 mM EDTA, 130 mM NaCl, 1% Triton X-100, 10 mg/ml PMSF, 10 mg/ml aprotinin, 10 mg/ml leupeptin, 5 mM phenanthroline, and 28 mM benzamidine-HCl) for 30 min on ice. The lysates were clearly centrifugation and then quantified using a modified BCA assay (Thermo Scientific). Each sample was separated with 12% sodium dodecyl sulfate-polyacrylamide gel electrophoresis, followed by electrotransfer onto the nitrocellulose membranes (Hybond-ECL; Amersham Pharmacia Biotech). The blots were blocked in 5% non-fat skim milk and incubated with a rabbit anti-AbOmpA immune serum and β-actin antibodies (Santa Cruz Biotechnology). The membranes were incubated with a secondary antibody coupled to horseradish peroxidase and developed using an enhanced chemiluminescence system (ECL plus; Amersham Pharmacia Biotech).

### Cell proliferation assay

The growth of cell treated with different rAbOmpA proteins was measured with a Premix WST1 cell proliferation assay system (TaKaRa Shuzo) [11]. The cells were seeded at a concentration of 2.0×10^5^/ml in a 96-well microplate. After treating with different concentrations of micelles for 24 h, cell growth was measured at 450 nm 3 h after treatment with WST1.

### Flow cytometric analysis

Cells were treated with OMVs for 24 h and stained with FITC-conjugated Annexin V and PI (BD Pharmingen) according to the manufacturer's instructions. The 1×10^6^ cells were stained with FITC-conjugated Annexin V in Annexin V binding buffer for 15 min. PI was added to determine alterations in cell membrane integrity. The samples were immediately analyzed by the flow cytometry and CellQuest Pro software (BD Biosciences). For each sample, 5,000 or 10^4^ cells were acquired for data analysis.

### Confocal microscopy

The cultured cells were seeded at a density of 5×10^4^ on glass coverslips the day before the assay. After treating the OMVs, the cells were washed in PBS, fixed in 4% paraformaldehyde and permeabilized for 10 min in PBS containing 0.25% Triton X-100. The OMVs were labeled with a polyclonal anti-rabbit AbOmpA antibody, followed by Alexa-488- or Alexa-568-conjugated goat anti-rabbit IgG antibody (Molecular Probes). The nuclei of the cells were stained with DAPI (Molecular Probes). HeLa cells were treated with 10 mM MβCD (Sigma-Aldrich) to disrupt cholesterol-rich membrane domains for 45 min in serum-free medium at 37°C in a CO_2_ incubator. After washing with PBS, cells were treated with OMVs. The samples were observed with a Carl-Zeiss confocal fluorescent microscope.

### Determination of β-lactamase activity

The chromogenic cephalosporin nitrocefin (Oxoid, U. K) was used to determine β-lactamase activity [43]. The assay was performed at 30°C with 100 mM nitrocefin in PBS (pH 7.4). Hydrolysis was monitored at 480 nm using a UV spectrophotometer (Shimadzu, Japan).

### TEM analysis

After the resuspension of OMV preparations in PBS, the samples were applied to copper grids and stained with 2% uranyl acetate. The lung tissues removed from the infected mice were fixed with 2.5% glutaraldehyde and post-fixed in 1% osmium tetroxide. The samples were subsequently dehydrated in a series of ethanol concentrations and embedded in Epon. Thin sections were cut with an ultramicrotome (RMC Boeckeler Instruments) equipped with a diamond knife and stained with 3% uranyl acetate and lead citrate. The samples were then visualized with a TEM (Hitachi H-7500, Hitachi, Japan) operated at 120 kV.

### Mouse pneumonia model

Eight-week-old female C57BL/6 mice were maintained under specific pathogen free conditions. Immunocompromised mice were infected with *A. baumannii* ATCC 19606^T^ [9]. Neutropenic mice were induced via intraperitoneal injections of cyclophosphamide (150 mg/Kg) on days -4 and -3 before bacterial injection [44]. The mice were anesthetized with pentobarbital and then 100 µl of 1×10^8^ cfu/ml of bacteria were administrated intratracheally. The control mice were injected with 100 µl of PBS (pH 7.4). The mice were sacrificed two days after bacterial challenge and the lungs were removed. All procedures involving animals were approved by the Animal Care Committee of Kyungpook National University (KNU2010-40).

## Supporting Information

Table S1 **Proteins identified in the OMV fraction of** ***A. baumannii*** **ATCC 19606^T^ using 1-DE and LC-MS/MS analysis.**
(XLS)
